# Pregnancy‐Induced Hypertension Complicated by Posterior Reversible Encephalopathy Syndrome: A Case Series of Two Patients

**DOI:** 10.1002/ccr3.73800

**Published:** 2026-11-06

**Authors:** Xintong Zhao, Mengqing Yan, Feixiang Long, Androniks Mumdzjans, Rongrong Song

**Affiliations:** ^1^ Graduate School of Bengbu Medical College Bengbu China; ^2^ Bengbu Medical College Bengbu China; ^3^ Department of Radiology Xuancheng People's Hospital Xuancheng China; ^4^ Department of Obstetrics and Gynecology Hull University Teaching Hospitals NHS Trust Hull UK; ^5^ Department of Obstetrics and Gynecology Xuancheng People's Hospital Xuancheng China

**Keywords:** case report, eclampsia, MRI, posterior reversible encephalopathy syndrome (PRES), severe preeclampsia

## Abstract

Posterior reversible encephalopathy syndrome (PRES) is a rare but potentially life‐threatening neurological complication associated with hypertensive disorders of pregnancy. This report presents two cases of pregnancy‐induced hypertension complicated by PRES, both clinically characterized by headache and visual disturbances, with characteristic neuroimaging findings. The clinical manifestations, laboratory findings, and magnetic resonance imaging (MRI) characteristics of two patients are described. Following prompt multidisciplinary consultation and immediate interventions, including strict blood pressure control, seizure prophylaxis, infection prevention, and appropriate obstetric management, both patients achieved favorable clinical outcomes. No significant neurological abnormalities were observed at the 42‐day postpartum follow‐up. In patients with severe pregnancy‐induced hypertension, PRES warrants recognition as a potential complication; management should prioritize rigorous blood pressure control, seizure prophylaxis, alleviation of pregnancy‐related physiological stress, and timely neuroimaging to guide treatment, with strategies balancing maternal and fetal safety—early recognition and intervention underpinned by standardized protocols and multidisciplinary collaboration being critical to improving prognosis and reducing complications.

## Key Clinical Message

Hypertension with headache, dizziness, and visual disturbance should raise suspicion for posterior reversible encephalopathy syndrome; prompt neuroimaging and multidisciplinary management are essential to prevent irreversible neurological injury.

## 1 Introduction

Hypertensive disorders of pregnancy (HDP) affect about 10% of pregnancies and include chronic hypertension, gestational hypertension, and preeclampsia. Poorly controlled BP increases risks of hypertensive crisis, eclampsia, stroke, and maternal/fetal death. Oral antihypertensives—such as methyldopa, labetalol, and nifedipine—are key to prolonging gestation and reducing complications [1]. However, a critical but often overlooked issue is that treatment success depends not only on drug selection but also on structured follow‐up and timely dose adjustment. Without these, persistent uncontrolled BP allows adverse outcomes to accumulate. Posterior reversible encephalopathy syndrome (PRES) is a rare but severe neurological complication of HDP, characterized by headache, seizures, visual disturbances, and altered mental status, with imaging showing vasogenic edema in bilateral parieto‐occipital regions [2]. By analyzing two typical cases, this article emphasizes the critical role of early identification, timely radiological evaluation, and multidisciplinary management in improving maternal and neonatal outcomes, so as to provide a reference for clinical practice.

## 2 Case Presentation

### 2.1 Case 1

#### 2.1.1 Case History/Examination

A 27‐year‐old woman presented at 27 + 5 weeks of gestation with acute onset of headache, seizures, and visual impairment lasting approximately 2 h, following 27 + 5 weeks of amenorrhea. On September 29, 2024, two frozen embryos (Day‐5 blastocysts) had been transferred at Nanjing Maternal and Child Health Hospital. Post‐transfer, she received fenmetramide, dexamethasone, progesterone, and other medications for luteal support.

At 12 + 1 weeks of gestation, she was hospitalized at our institution for severe pregnancy‐related vomiting and received blood pressure monitoring, intravenous fluid therapy, antiemetic treatment, potassium supplementation, and antihypertensive therapy. During hospitalization, her blood pressure ranged from 122–140/81–99 mmHg, and she was prescribed oral labetalol (50 mg every 8 h) and enteric‐coated aspirin (0.1 g daily). After discharge, home blood pressure monitoring revealed values fluctuating between 130–140/90–100 mmHg. At 18 + 5 weeks of gestation, routine antenatal examination at our institution revealed proteinuria and suboptimal blood pressure control. Accordingly, her antihypertensive regimen was adjusted: labetalol 150 mg three times daily was initiated, while aspirin was continued at the previous dose. Subsequent self‐monitored blood pressure measurements ranged from 110–120/95–99 mmHg.

#### 2.1.2 Methods

The patient reported generalized edema for nearly 3 days prior to the current admission. At 27 + 5 weeks of gestation, she experienced sudden visual disturbance upon awakening, accompanied by severe headache and a single generalized seizure lasting approximately 1 min, as reported by family members. She was transported to the emergency department, where her blood pressure was measured at 218/128 mmHg.

Immediate management included oral nifedipine (20 mg), intravenous magnesium sulfate for seizure control, continuous electrocardiographic monitoring, fetal heart rate monitoring, and oxygen supplementation. Despite treatment, her blood pressure remained markedly elevated, prompting initiation and continuation of intravenous urapidil infusion. Cranial computed tomography (CT) demonstrated symmetrical patchy low‐density areas in both occipital lobes with indistinct margins and mildly blurred adjacent sulci, suggestive of posterior reversible encephalopathy syndrome (PRES). Mild thickening of the posterior nasopharyngeal wall soft tissue was also noted.

Following multidisciplinary consultation, a diagnosis of eclampsia was established, and given the risk of further neurological deterioration, emergency cesarean delivery under general anesthesia was recommended. The procedure was performed uneventfully, resulting in the live birth of a male infant weighing 1050 g. The 1‐min Apgar score was 2, with 1 point each for heart rate and reflex irritability. Immediately after umbilical cord clamping, the neonate received warmth, airway clearance, and endotracheal intubation, and was connected to a transport ventilator before being transferred to the neonatology department. After admission to the neonatal department, the neonate was treated with mechanical ventilation, caffeine for respiratory stimulation, intratracheal instillation of pulmonary surfactant, anti‐infective therapy with cefotaxime sodium combined with penicillin, vitamin K1 for bleeding prevention, plasma transfusion, red blood cell transfusion, and nutritional fluid support. After 8 days of treatment, cranial ultrasonography indicated intraparenchymal hemorrhage, and the condition was critical. At the family's request, the patient was transferred to a higher‐level hospital for further diagnosis and treatment. On follow‐up, a drainage procedure for hydrocephalus was performed at the higher‐level hospital. At 7 months of age (November 19, 2025), the patient visited the pediatric department for treatment of bronchopneumonia and post‐hydrocephalus‐surgery status, and was cured and discharged. Currently, the patient is under follow‐up, with a generally good condition.

Postoperatively, the patient exhibited blurred vision, drowsiness, sluggish pupillary light reflexes, and mild cyanosis of the lips and mucous membranes. She was transferred to the intensive care unit (ICU) for close monitoring. Comprehensive laboratory evaluations revealed no significant autoimmune abnormalities. Management included airway maintenance, oxygen therapy, magnesium sulfate, urapidil infusion, mannitol for intracranial pressure reduction, prophylactic antibiotics, albumin infusion for hypoalbuminemia, and correction of electrolyte imbalances.

Over subsequent days, the patient demonstrated gradual clinical improvement. MRI and MRA performed on Postoperative Day 4 revealed symmetrical abnormal signals in both occipital lobes consistent with PRES (Figure 1). Follow‐up ophthalmologic examination identified bilateral exudative retinal detachment involving the macula.

**FIGURE 1:**
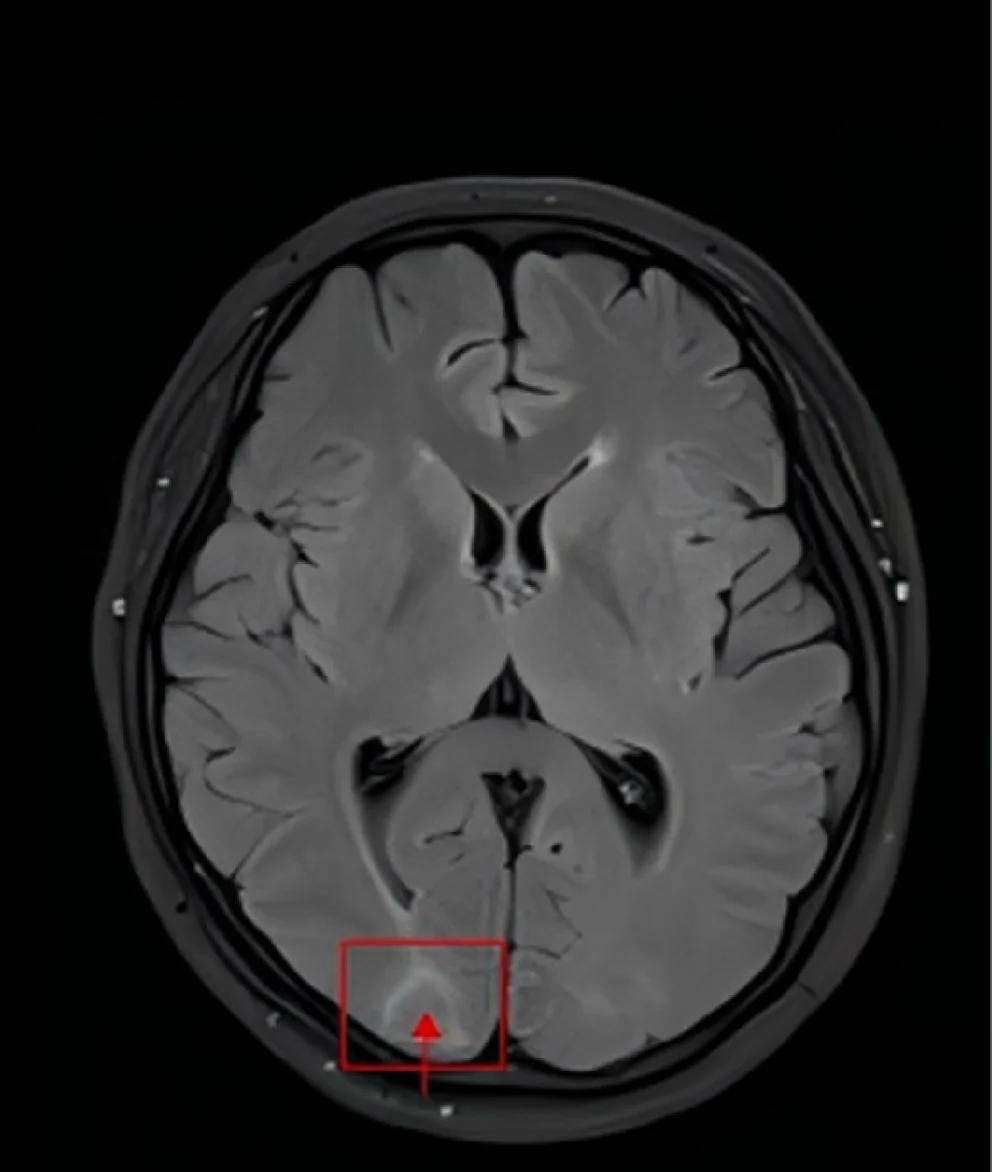
T2WI FLAIR.

#### 2.1.3 Outcome and Follow‐Up

The patient was discharged on Postoperative Day 9 in stable condition. At 42‐day postpartum follow‐up, all clinical and laboratory parameters had normalized. Repeat cranial MRI at 52 days postpartum showed complete resolution of previous abnormalities.

### 2.2 Case 2

#### 2.2.1 Case History/Examination

A 27‐year‐old primigravida at 37 + 3 weeks of gestation presented with a 24‐h history of intermittent epigastric pain, progressive dizziness, headache, and visual disturbance. During her pregnancy, the patient underwent regular and standard antenatal care at our institution, with consistently normal blood pressure recordings. She did not receive any medication throughout the gestation, and no pregnancy‐associated complications were observed.

#### 2.2.2 Methods

On admission, blood pressure was 165/106 mmHg, and oxygen saturation was 91%. Immediate management included oral nifedipine (20 mg), intravenous magnesium sulfate, and continuous maternal–fetal monitoring. Cranial CT showed no acute intracranial abnormalities. Given the progression of severe preeclampsia with neurological symptoms, emergency cesarean delivery was performed following multidisciplinary consultation.

Postoperative laboratory findings revealed severe anemia, thrombocytopenia, leukocytosis, and hypoalbuminemia, consistent with a HELLP‐syndrome variant. Brain MRI subsequently demonstrated parieto‐occipital subcortical hyperintensities characteristic of PRES (Figure 2).

**FIGURE 2:**
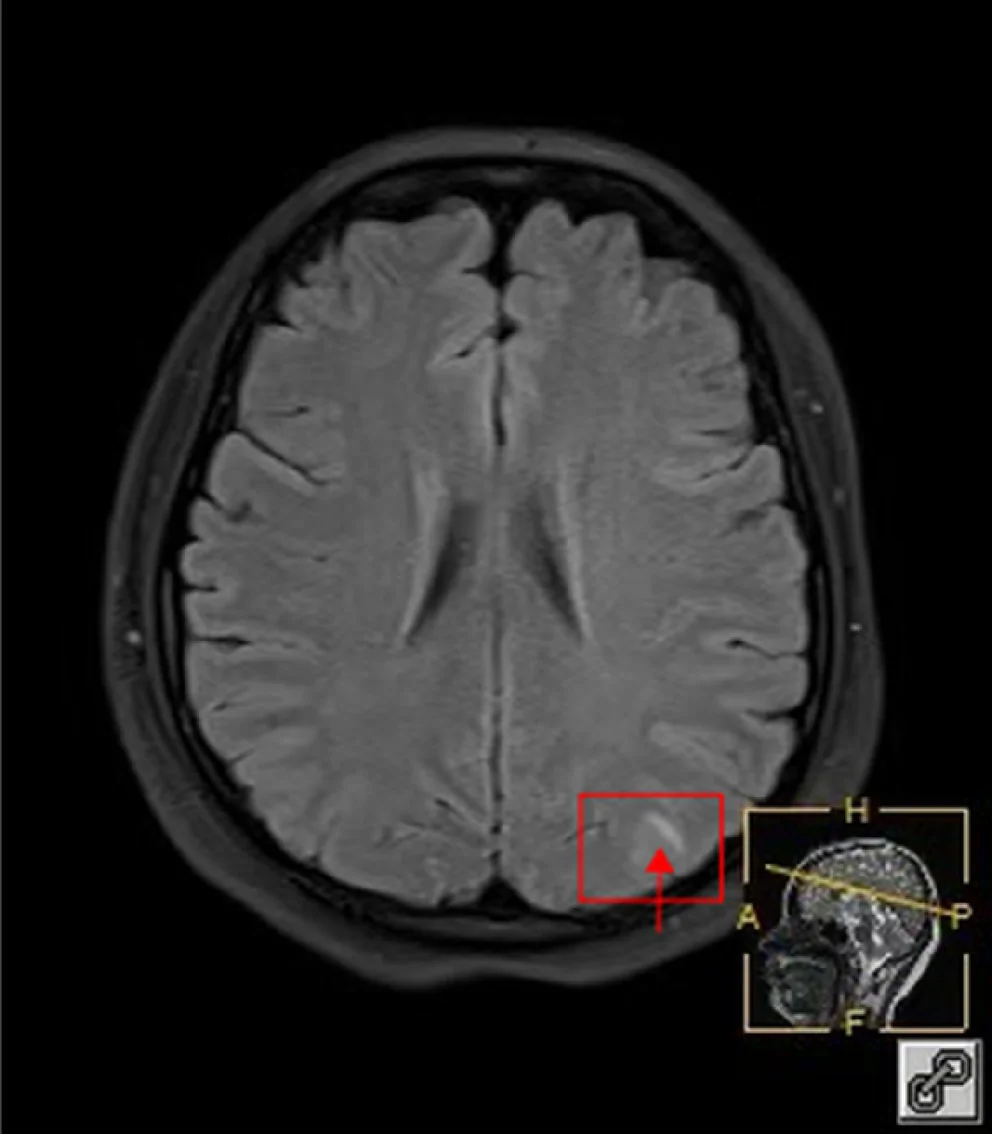
T2WI FLAIR.

#### 2.2.3 Outcome and Follow‐Up

The patient received comprehensive supportive treatment and was discharged on Postoperative Day 7. At 6‐week postpartum follow‐up, laboratory parameters had returned to normal. The patient declined repeat neuroimaging despite medical advice.

## 3 Discussion

Eclampsia is often associated with severe fetal–maternal circulatory imbalance, impaired placental perfusion, preterm birth, intrauterine growth restriction (IUGR), and an increased risk of intrauterine fetal death (IUFD). It is considered a major cause of preterm birth, low birth weight, and increased NICU admission rates [3]. Eclampsia represents a critical progression involving central nervous system dysfunction and may result in severe maternal and neonatal complications.

Reversible posterior leukoencephalopathy syndrome (PRES), first described by Hinchey et al. in 1996, is characterized by acute neurological symptoms and distinctive imaging findings [4]. Symptoms often develop rapidly over a few hours and reach their most severe form within 12–48 h. Symptoms typically resolve within 1 week, but may persist longer [5]. Studies have shown that autoimmune diseases, severe hypertension, organ transplantation, cytotoxic drugs, sepsis, and immunosuppressive therapy are all associated with the development of reversible posterior leukoencephalopathy syndrome [6]. Imaging plays a crucial role in the diagnosis and assessment of PRES, providing key clinical information to help clarify the etiology and guide treatment strategies. MRI is the most valuable diagnostic tool, with typical findings showing slightly low signal intensity on T1‐weighted images (T1WI), high signal intensity on T2‐weighted images (T2WI) and FLAIR images, and slightly low signal intensity on diffusion‐weighted imaging (DWI) with a high apparent diffusion coefficient (ADC). On FLAIR MRI, PRES is typically characterized by edema in the occipital and parieto‐occipital white matter. Both DWI and ADC sequences show isointensity, indicating vasogenic cerebral edema, with some lesions exhibiting slightly higher signals on DWI due to the T2 penetration effect [7]. Preeclampsia is a severe pregnancy‐specific multisystem disorder associated with significant maternal and perinatal morbidity and mortality, affecting approximately 2%–8% of pregnancies worldwide with notable geographic variations [8]. Although the exact pathogenesis of preeclampsia remains incompletely understood, it is widely regarded as a syndrome resulting from the complex interplay of multiple factors, mechanisms, and pathways [9]. While pharmacological interventions can alleviate symptoms, their efficacy is often limited and accompanied by potential adverse effects, posing dual risks to both the mother and fetus [10]. In patients with eclampsia or severe preeclampsia, PRES is an important but often underrecognized complication. Neuroimaging, particularly MRI, plays a pivotal role in diagnosis.

We report two cases of hypertensive disorders of pregnancy (HDP) with posterior reversible encephalopathy syndrome (PRES) presenting distinct clinical trajectories. Four critical lessons emerge. First, PRES pathogenesis is not determined by absolute blood pressure (BP) alone. Case 1 had chronic hypertension; Case 2 was normotensive until acute onset at 37 weeks. Both developed PRES, suggesting that rate of BP rise and endothelial vulnerability—particularly HELLP‐associated microangiopathy—outweigh chronic BP levels in triggering blood–brain barrier disruption. Second, Case 1 identifies assisted reproductive technology as a potential predisposing factor for early‐onset severe preeclampsia and PRES, warranting intensified neurologic surveillance. Third, pharmacologically “controlled” diastolic BP (95–99 mmHg) in Case 1 failed to prevent eclamptic seizure, cautioning against over‐reliance on home BP monitoring and emphasizing symptom‐based vigilance. Fourth, divergent follow‐up (imaging resolution in Case 1 vs. patient refusal in Case 2) highlights that clinical recovery does not guarantee radiological reversibility; postpartum MRI at 6–12 weeks is advisable.

## 4 Conclusion

Both cases presented here highlight persistent severe hypertension as a key precipitating factor for PRES. Early recognition, prompt neuroimaging, aggressive blood pressure control, seizure prophylaxis with magnesium sulfate, and timely delivery were essential in achieving favorable outcomes. Eclampsia and severe preeclampsia represent the most critical manifestations of hypertensive disorders in pregnancy. When complicated by PRES, vigilant neurological monitoring, timely imaging evaluation, and coordinated multidisciplinary management are imperative. Early diagnosis and individualized, guideline‐based treatment can lead to complete neurological recovery and improved maternal and neonatal outcomes.

## Author Contributions

**Xintong Zhao:** conceptualization, writing – original draft, writing – review and editing, validation. **Mengqing Yan:** writing – original draft, writing – review and editing, validation. **Feixiang Long:** writing – review and editing, writing – original draft. **Androniks Mumdzjans:** writing – review and editing. **Rongrong Song:** supervision, writing – review and editing, validation, visualization.

## Funding

The authors have nothing to report.

## Ethics Statement

The institutional board review at Xuancheng People's Hospital granted approval for the study is available upon request from the corresponding author (ethics approval code: AF‐SOP‐29‐1.0).

## Consent

Written informed consent from the patients was obtained according to journal guidelines.

## Conflicts of Interest

The authors declare no conflicts of interest.

## Data Availability

The data that support the findings of this study are available on request from the corresponding author. The data are not publicly available due to privacy or ethical restrictions.
